# Supplementary material for: Allosteric MAPKAPK2 inhibitors improve plaque stability in advanced atherosclerosis
Source: PLoS One. 2021 May 13;16(5):e0246600. doi: 10.1371/journal.pone.0246600 (PMC8118275; doi:10.1371/journal.pone.0246600)

Figure S1B- 1st panel

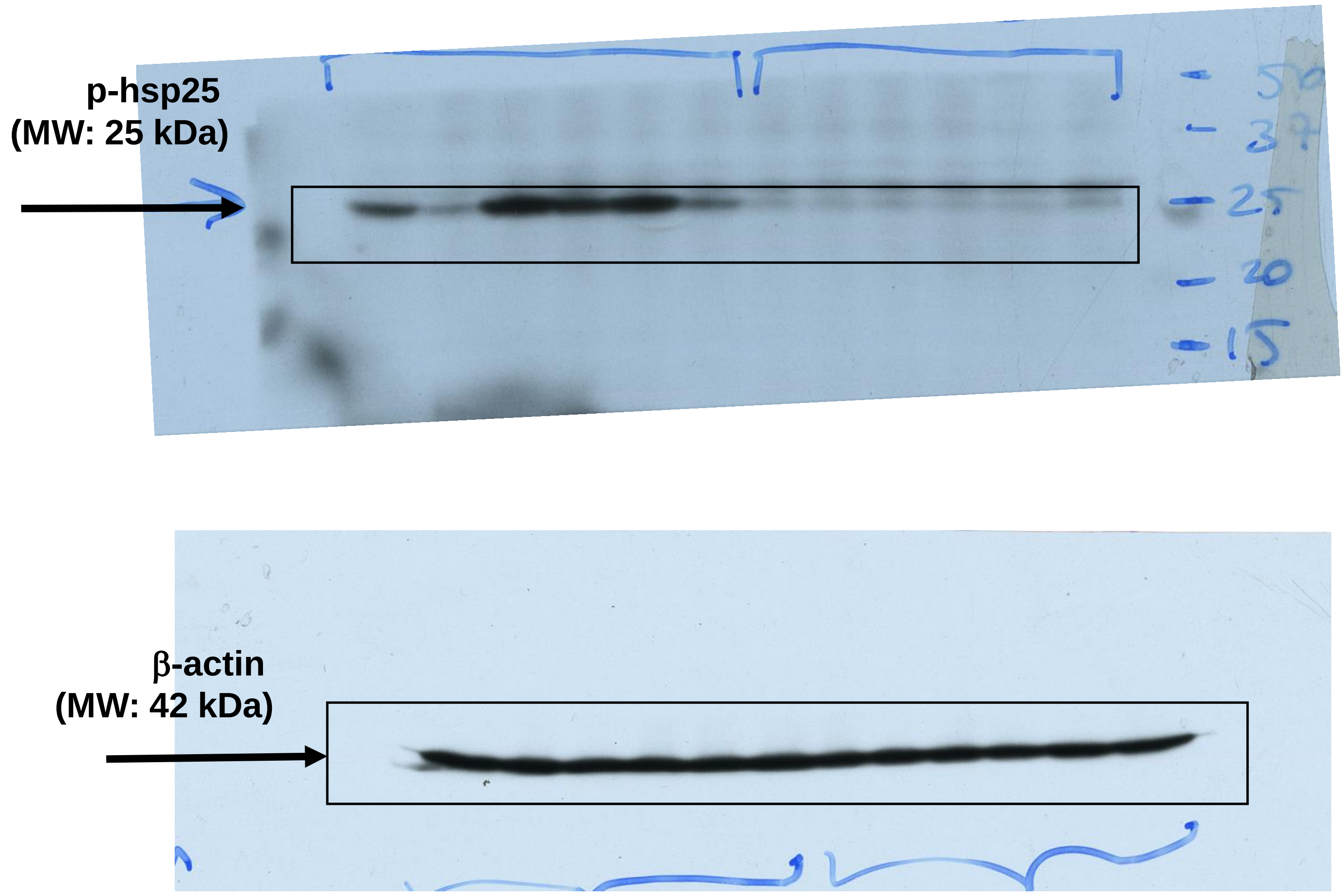

Figure S1B- 2nd panel

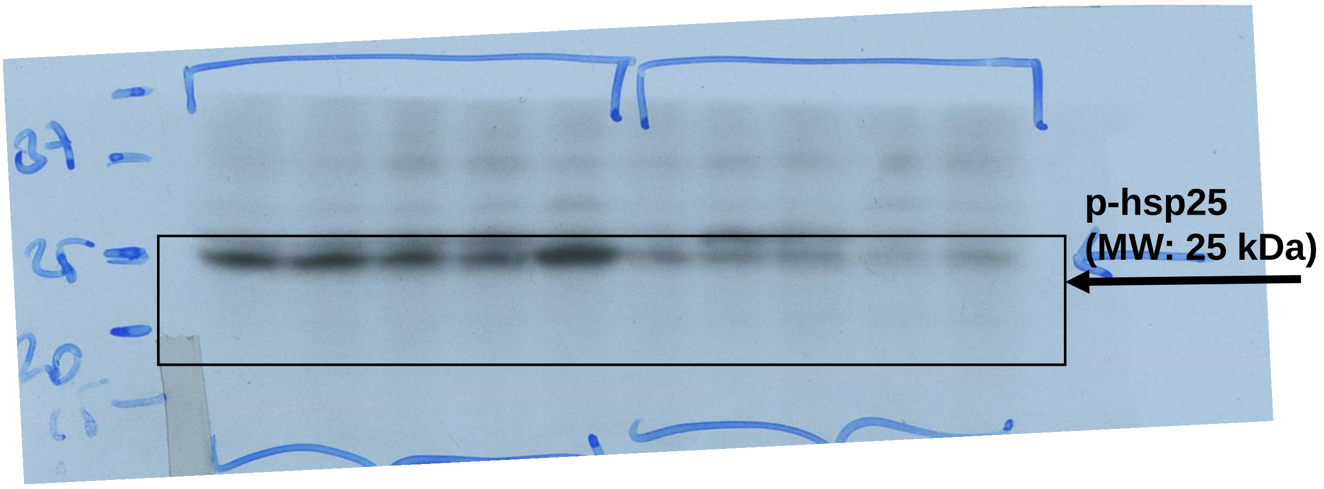

Figure S1B- 2nd panel

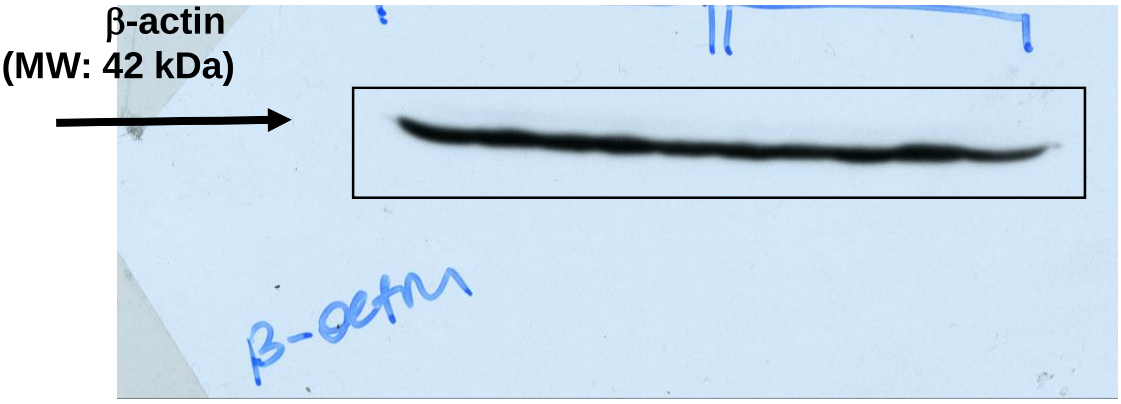

Figure S1B- 3rd panel

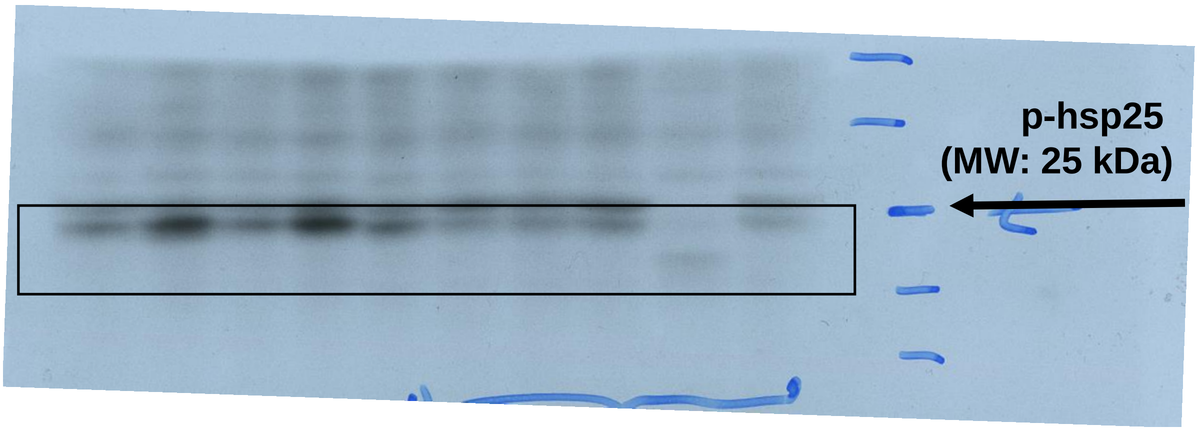

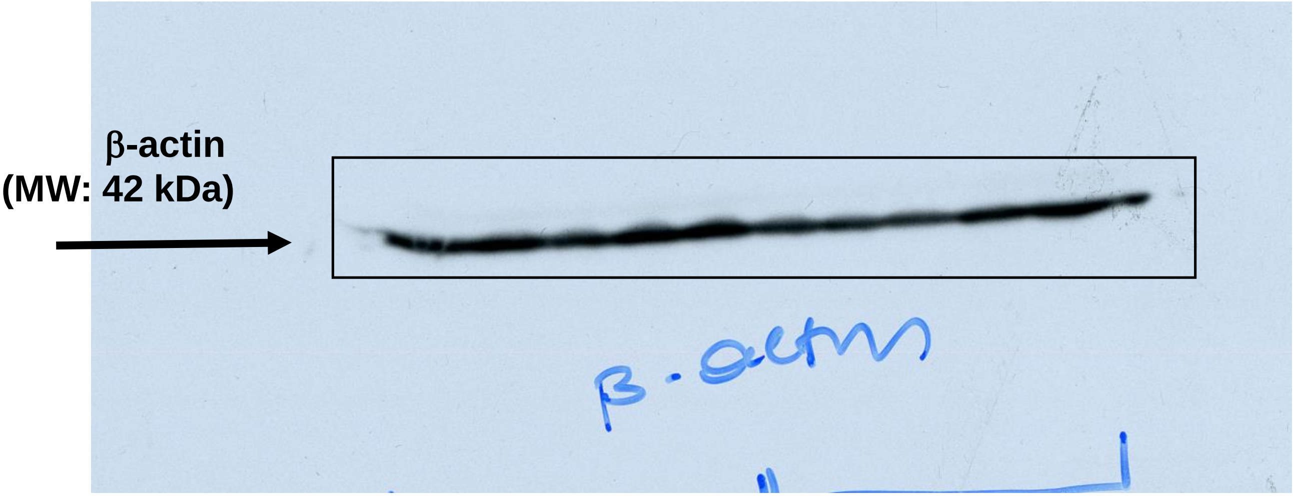

Supplement: S1 Raw images — (PDF) [file pone.0246600.s001.pdf]
