# Supplementary material for: Allosteric MAPKAPK2 inhibitors improve plaque stability in advanced atherosclerosis
Source: PLoS One. 2021 May 13;16(5):e0246600. doi: 10.1371/journal.pone.0246600 (PMC8118275; doi:10.1371/journal.pone.0246600)
Supplement: S1 File — (PDF) [file pone.0246600.s002.pdf]

Fig S1

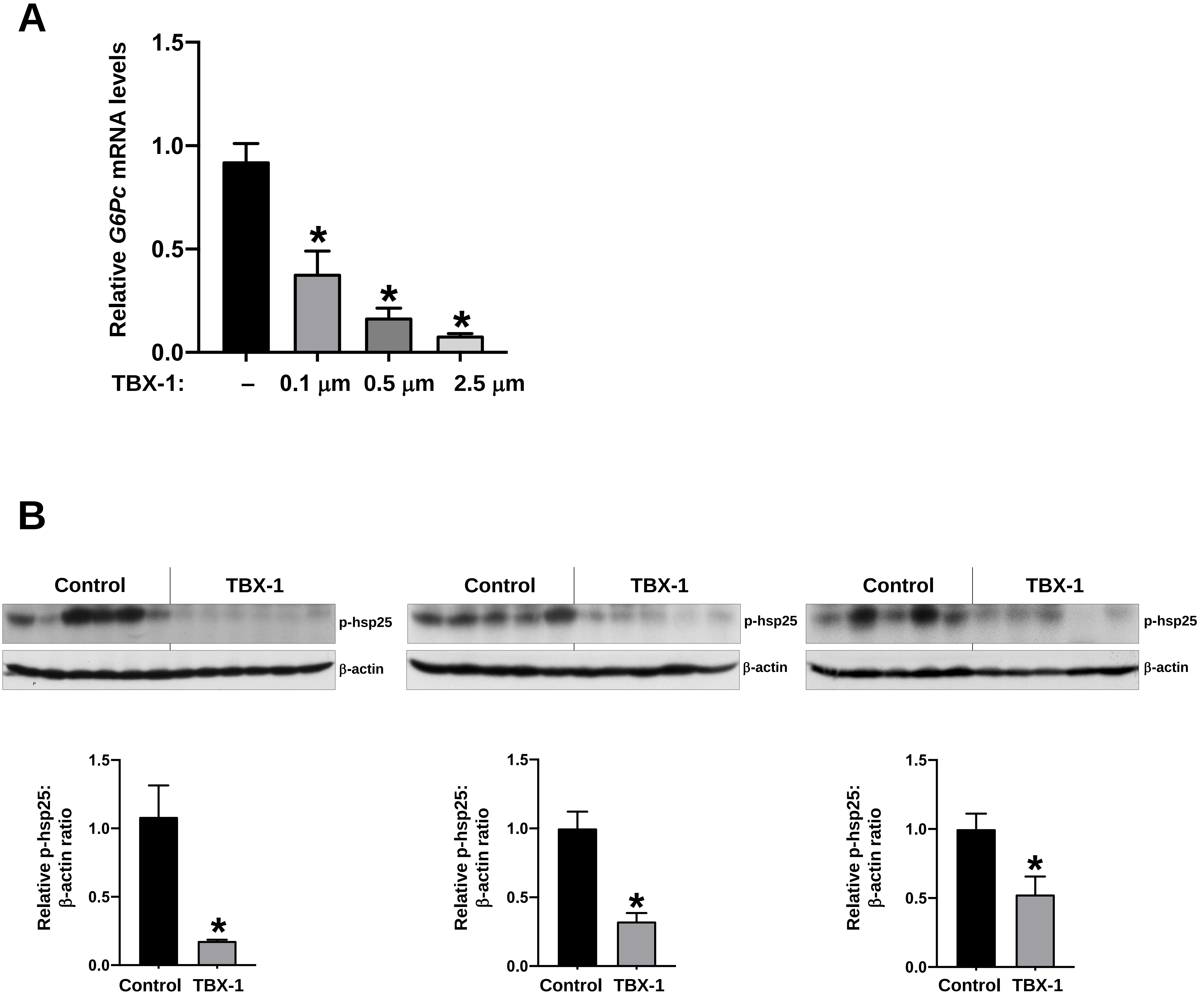
A
Relative G6Pc mRNA levels
1.5
1.0
0.5
0.0
*
TBX-1:
–
0.1 μm
0.5 μm
2.5 μm
B
Control
TBX-1
p-hsp25
β-actin
Relative p-hsp25:
β-actin ratio
Control
TBX-1

Fig S2

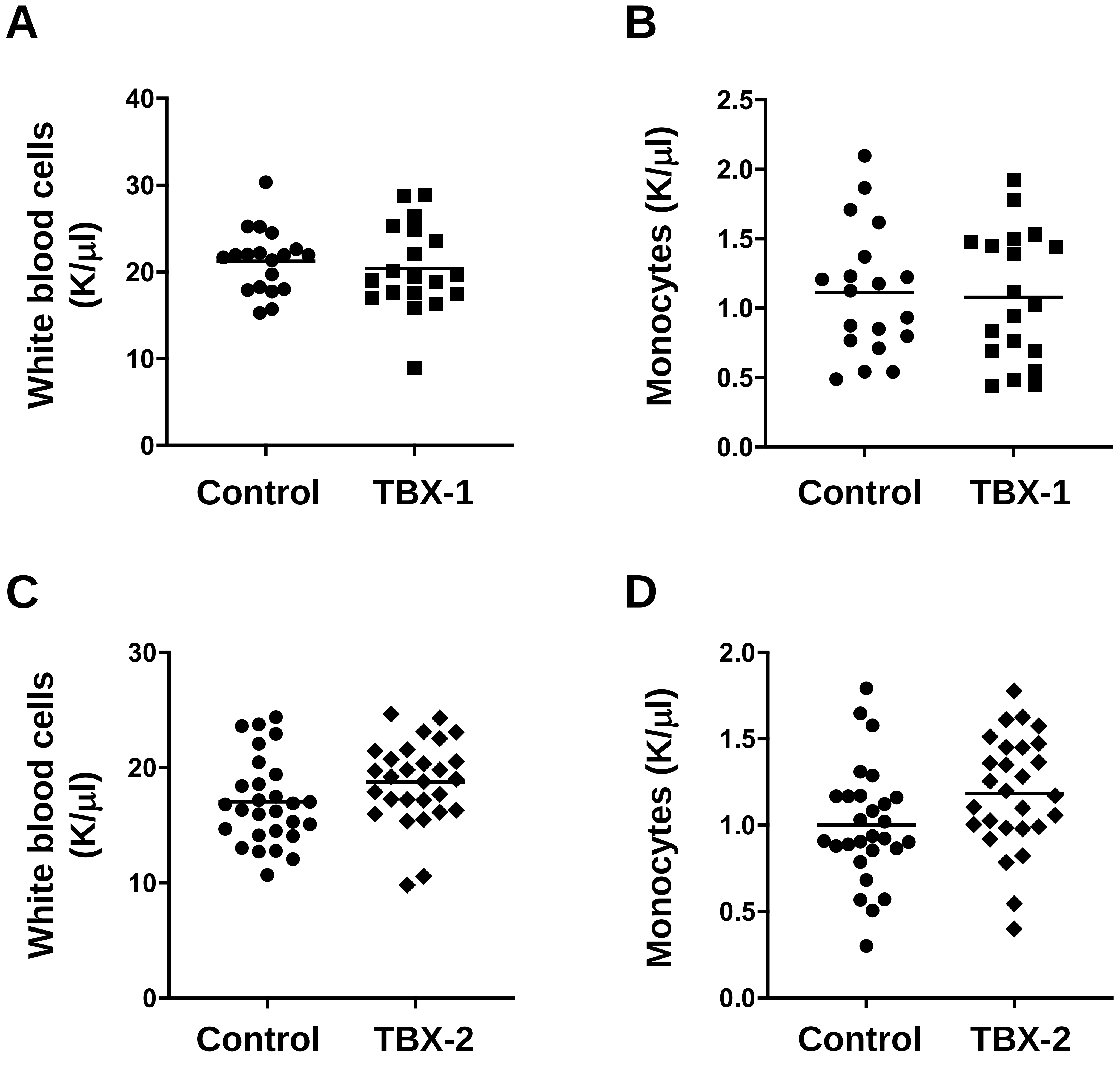
A
White blood cells (K/µl)
40
30
20
10
0
Control
TBX-1
B
Monocytes (K/µl)
2.5
2.0
1.5
1.0
0.5
0.0
Control
TBX-1
C
White blood cells (K/µl)
30
20
10
0
Control
TBX-2
D
Monocytes (K/µl)
2.0
1.5
1.0
0.5
0.0
Control
TBX-2

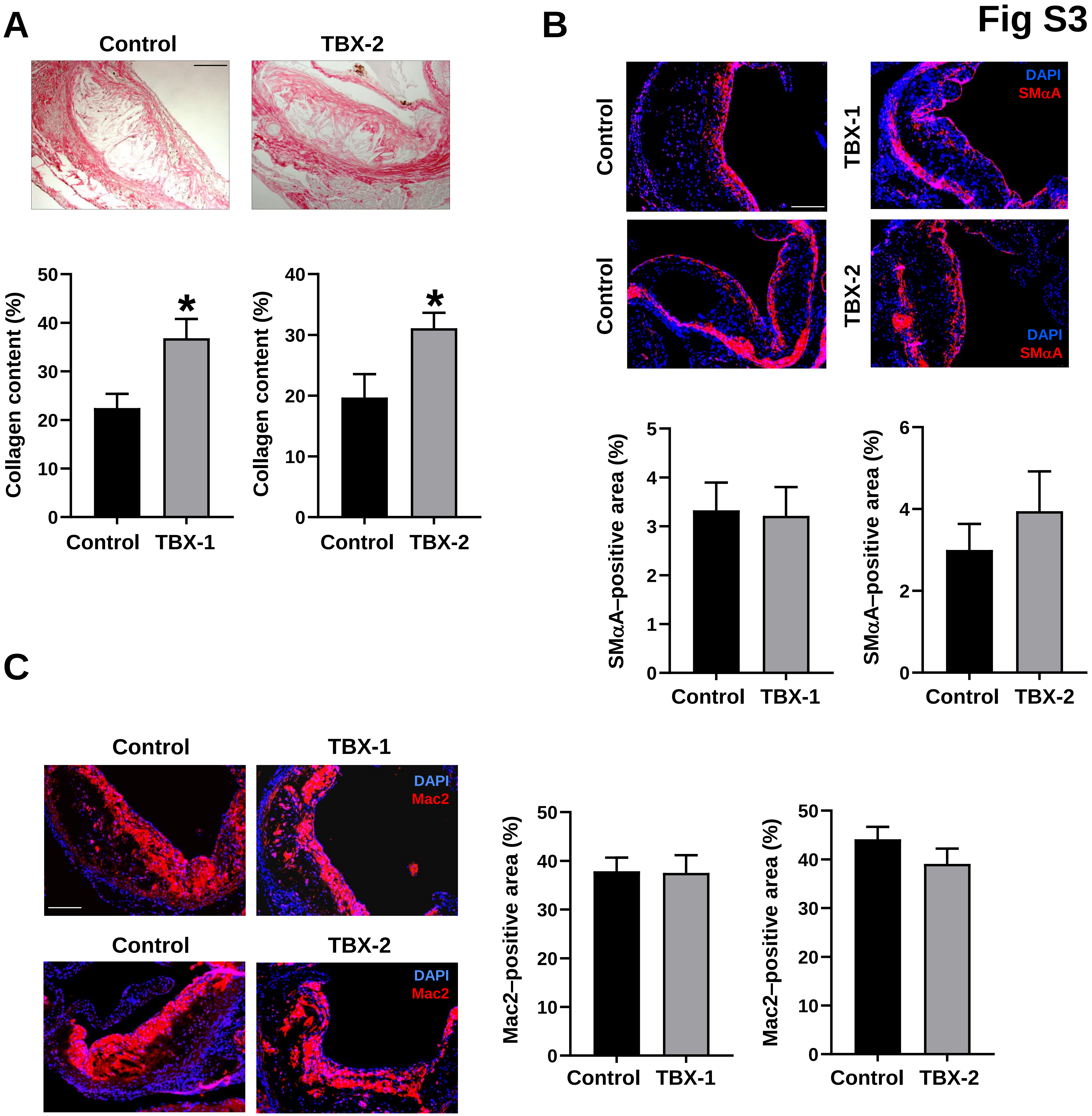

Fig S3
A
Control
TBX-2
Collagen content (%)
Control
TBX-1
Control
TBX-2
B
Control
TBX-1
Control
TBX-2
DAPI
SMαA
SMαA–positive area (%)
Control
TBX-1
Control
TBX-2
C
Control
TBX-1
Control
TBX-2
DAPI
Mac2
Mac2–positive area (%)
Control
TBX-1
Control
TBX-2

Supporting information

S1 Fig. MK2 inhibitor reduces *G6Pc* mRNA in isolated hepatocytes and lowers p-hsp25 levels in WD-fed *Ldlr*$^{-/-}$ mice liver. (**A**) Primary mouse hepatocytes were treated with the indicated concentrations of TBX-1 for 1 h, followed by treatment with TBX-1 and forskolin for 4 h in serum-free media. RNA was assayed for *G6pc* mRNA by RT-qPCR (n = 3; mean ± SEM, $p < 0.001$). (**B**) Liver extracts from three different randomly selected subsets of TBX-1–treated *Ldlr*$^{-/-}$ mice were assayed for phospho-hsp25 (p-hsp25) and β-actin by immunoblot. Densitometric quantification of the data are shown in the bar graphs (n = 5-6 mice/group; mean ± SEM, p = 0.003, p = 0.001 and p = 0.02, respectively).

S2 Fig. Blood leukocyte and monocyte counts are similar between control and MK2 inhibitor–treated mice. Mice were treated as in Figure 1. Whole blood was analyzed for the number of total white blood cells (**A**) and monocytes (**B**) from a randomly selected subset of TBX-1–treated *Ldlr*$^{-/-}$ mice (n = 19 mice/group; mean ± SEM). (**C-D**) Same as in (A-B) except that the blood of TBX-2–treated mice was assayed (n = 28 mice/group; mean ± SEM).

S3 Fig. Lesional collagen content is increased, whereas macrophage and smooth muscle cell numbers are similar between control and MK2 inhibitor–treated mice. Mice were treated as in Figure 1. (**A**) Aortic root sections from the indicated groups of mice were stained with picrosirius red to visualize the collagen content. Scale bar: 100 μm. Picrosirius red staining was quantified, and collagen content is expressed as percent of total lesion area (n = 7-10 mice/group; mean ± SEM, p = 0.009 and p = 0.03, respectively). (**B-C**) Aortic root sections from the indicated groups of mice were stained with Mac2 (red), smooth muscle α-actin (SMαA, red), and DAPI (blue) to visualize macrophages and smooth muscle cells, respectively. Scale bar:

100 μm. Mac2– and SMαA–positive areas were quantified and expressed as percent of total lesion area (n = 7-10 mice/group; mean ± SEM).

Supplementary methods

Synthesis of MK2 inhibitors

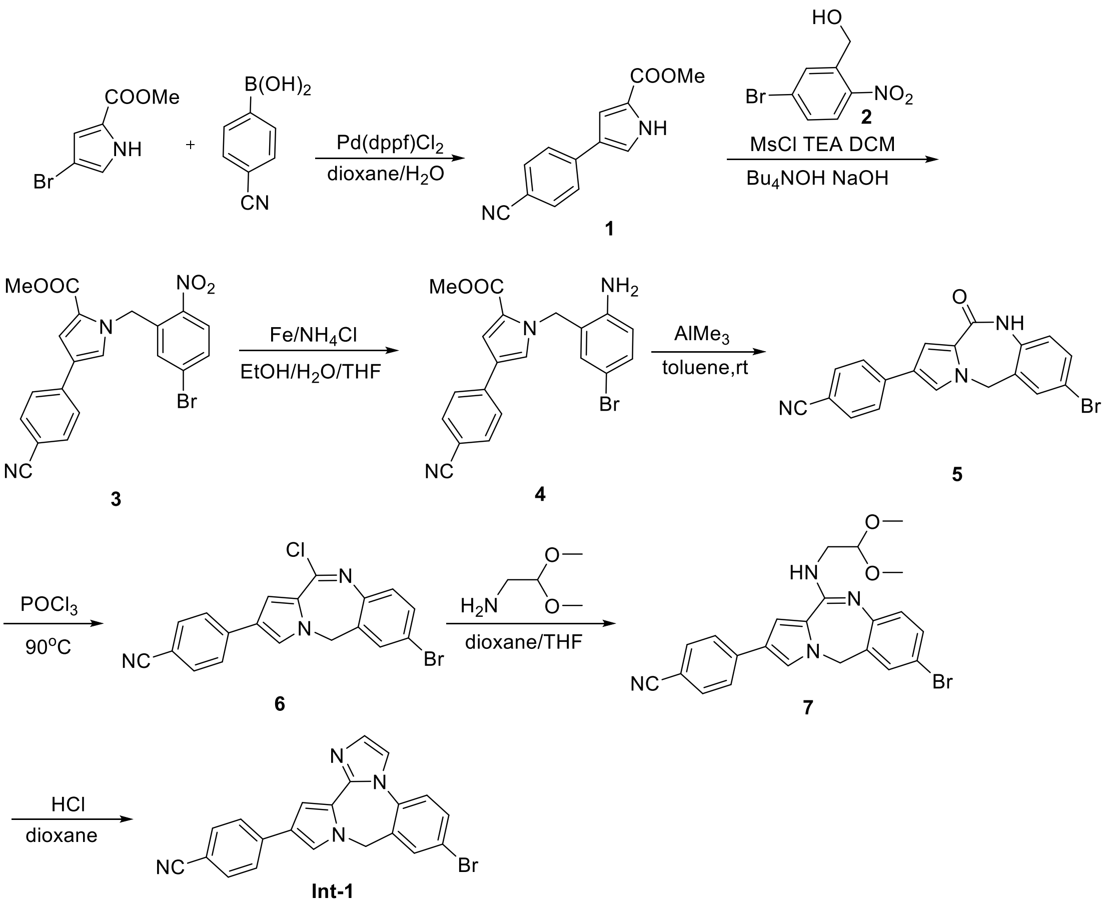

Procedures:

<u>Methyl 4-(4-cyanophenyl)-1H-pyrrole-2-carboxylate (1)</u>

A suspension of methyl 4-bromo-1H-pyrrole-2-carboxylate (100 g, 489 mmol, 1.0 eq), ((4-cyanophenyl)boronic acid (108 g, 734.6 mmol, 1.5 eq), K_2CO_3 (202 g, 1467 mmol, 3.0 eq),

$Pd(dppf)Cl_2$ (17.9 g, 24.5 mmol, 0.05 eq) in dioxane/H_2O (1.8 L, 5:1) was de-gassed and then heated to 100 °C for 20 h under N_2. The reaction mixture was cooled down to room temperature and poured into iced water (2L) with stirring. The precipitate formed was collected by filtration, which was re-dissolved in DCM (~3 L) and washed with brine. The residue was loaded onto a short silica gel column and flashed with PE: DCM (1:1). The product-containing fractions were combined and concentrated under reduced pressure. The residue was re-dissolved in DCM (~1 L), and 10 L of petroleum ether was slowly added to precipitate the product. After stirring for 1h, the solid was collected by filtration and dried to give desired product (92 g, 93% purity) as a pale yellow solid: ^1H NMR (400MHz, DMSO-d_6) δ12.30 (br. s., 1H), 7.84 (d, J =8.4 Hz, 2H), 7.79 - 7.69 (m, 3H), 7.33 (br. s., 1H), 3.80 (s, 3H). ESI $[M+H]^+$ = 227.1

(5-bromo-2-nitrophenyl) methanol (2)

To a solution of 5-bromo-2-nitrobenzaldehyde (250 g, 1.086 mol, 1.0 eq), $NaBH_4$ (12.4 g, 326 mmol, 0.3 eq) in MeOH (1.0 L) was added batch-wise over 30 minutes at 0 °C. After addition, the mixture was allowed to warm to room temperature and continued to stir for 10 min. The mixture was then quenched by slowly addition of sat. NH_4Cl (1L) and concentrated under reduced pressure to remove volatile solvent. The residue was extracted with EA (1Lx3), and the combined organic layers were washed with brine, dried over Na_2SO_4, then concentrated under reduced pressure to give the desired product (252 g, 1086 mmol) as a white solid: ^1H NMR (400MHz, $CDCl_3$) δ 7.98 - 7.91 (m, 2H), 7.55 (dd, J =1.8, 8.8 Hz, 1H), 4.97 (br. s., 2H), 2.77 (br. s, 1H). ESI $[M+H]^+$ = 232.2/234.2

Methyl 1-(5-bromo-2-nitrobenzyl)-4-(4-cyanophenyl)-1H-pyrrole-2-carboxylate (3)

To a solution of compound **2** (105 g, 442.5 mmol, 1.0 eq), MsCl (37.6 mL, 486.7 mmol, 1.1 eq) and TEA (123 mL, 885 mmol, 2.0 eq) in DCM (1.5 L) were added at 0 °C, and the mixture was stirred for 0.5 h at 20 °C. Compound **1** (100 g, 442.5 mmol, 1.0 eq) was added to the solution

followed by addition of tetrabutylammonium hydroxide (45 g, 44.2 mmol, 0.1 eq) and a solution of NaOH (2M, 1.1 L, 5.0 eq) at 0 °C. The mixture was then allowed to stir at room temperature for 2 h. The yellow precipitate was collected by filtration. The residue was added to EtOH (200 mL) and stirred for 1 h. The precipitate was collected by filtration to give the desired product (280 g, 636 mmol, 71.9% yield) as an off-white solid: ^{1}H NMR (400MHz, DMSO-d_6) δ 8.08 (d, *J* = 8.8 Hz, 1H), 8.00 (d, *J* = 1.8 Hz, 1H), 7.84 - 7.76 (m, 5H), 7.55 (d, *J* = 1.8 Hz, 1H), 6.71 (d, *J* = 1.3 Hz, 1H), 5.86 (s, 2H), 3.29 (s, 3H). ESI $[M+H]^+$ = 440.1/ 442.1

Methyl 1-(2-amino-5-bromobenzyl)-4-(4-cyanophenyl)-1H-pyrrole-2-carboxylate (4)

A suspension of compound **3** (186 g, 423.4 mmol, 1.0 eq), Fe (118.2 g, 2.1 mol, 5.0 eq) and NH_4Cl (226.5 g, 4.2 mol, 10.0 eq) in EtOH (2 L), H_2O (1 L) and THF (2 L) were heated to 80 °C for 2 h. The mixture was concentrated to dryness and the residue was re-dissolved in hot THF (20 L*5). The mixture was filtered through a pad of celite, and the filtrate was concentrated. The residue was slurried with EtOH (1L) and stirred for 1 h. The solid was collected by filtration to give the desired product (138 g): ^{1}H NMR (400MHz, DMSO-d_6) δ 7.88 (br. s., 1H), 7.85 - 7.68 (m, 4H), 7.50 (br. s., 1H), 7.09 (d, *J* = 7.9 Hz, 1H), 6.62 (d, *J* = 8.4 Hz, 1H), 6.33 (br. s., 1H), 5.37 (br. s., 4H), 3.73 (br. s., 3H). ESI $[M+H]^+$ = 410.2/412.2

4-(7-bromo-11-oxo-10,11-dihydro-5H-benzo[e]pyrrolo[1,2-a][1,4]diazepin-2-yl)benzonitrile (5)

To a suspension of compound **4** (100 g, 244 mmol, 1.0 eq), Me_3Al (2 M in toluene, 609 mL, 5.0 eq) in toluene (1L) was added dropwise at 0 °C, and the mixture was stirred for 10 h at 20 °C under N_2. The mixture was poured into 1M iced HCl (2M, 500 mL). The resulting mixture was filtered, and the filter cake was washed with $NaHCO_3$ (1 L). The solid was slurried with EtOH (700 mL) and stirred for 2 h and then collected by filtration. After drying under vacuum, the desired product was obtained (86.2 g, 166 mmol, 93.7% yield) as a yellow solid: ^{1}H NMR (400MHz,

DMSO-d_6) δ 10.30 (s, 1H), 7.73 (s, 4H), 7.66 (dd, J = 2.0, 4.2 Hz, 2H), 7.50 (dd, J = 2.2, 8.4 Hz, 1H), 7.27 (d, J = 1.8 Hz, 1H), 7.13 (d, J = 8.8 Hz, 1H), 5.21 (s, 2H). ESI $[M+H]^+$ = 378.2/380.2

4-(7-bromo-11-chloro-5H-benzo[e]pyrrolo[1,2-a][1,4]diazepin-2-yl)benzonitrile (6)

A solution of compound **5** (80 g, 211 mol, 1.0 eq) in $POCl_3$ (300 mL) was stirred for 18 h at 90 °C. The solution was concentrated under reduced pressure and the residue was dissolved in EA (3 L). The organic solution was poured into iced-$NaHCO_3$ solution (2L) and stirred for 30 min. The organic layer was separated and washed with brine, and dried over Na_2SO_4. After concentration under reduced pressure, the crude product (76 g, 93.7% yield) was used immediately to next step. ESI $[M+H]^+$ = 396.2/398.2

4-(7-bromo-9H-benzo[e]pyrrolo[1,2-a][1,2,4]triazolo[3,4-c][1,4]diazepin-12-yl)benzonitrile

To a solution of compound **6** (40 g, 88 mmol, 1.0 eq), formohydrazide (160 g, 2.66 mol, 30.0 eq) in THF/dioxane (1:1, 800 mL) was added, and the mixture was heated to 120 °C for 10 h. The mixture was cooled to room temperature. The precipitate was collected by filtration and further purified by trituration with MeOH and MTBE to give desired product (26 g) as a yellow solid. ESI $[M+H]^+$ = 402.1/404.1.

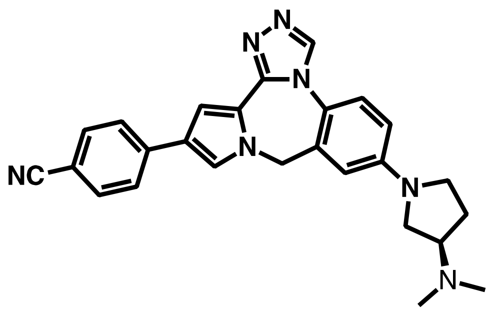

(R)-4-(7-(3-(Dimethylamino)pyrrolidin-1-yl)-9H-benzo[e]pyrrolo[1,2-a][1,2,4] triazolo[3,4-c][1,4]diazepin-12-yl)benzonitrile (TBX-1)

A suspension of the above intermediate (10 g, 2.8 mmol,1.0 eq), (R)-N,N-dimethylpyrrolidin-3-amine (5.7 g, 49.7mmol, 2.0 eq), BINAP(3.1 g, 4.97 mmol, 0.2 eq), $Pd_2(dba)_3$(2.3 g ,2.48 mmol, 0.1 eq), t-BuONa (7.1 g, 74.6mmol, 3.0 eq) in dry 1,4-dioxane (200 mL) were stirred at 95 °C under N_2 for 4 h. The reaction mixture was cooled to room temperature and concentrated under reduced pressure, and the resulting residue was purified by silica gel column (DMC: MeOH= 30:1-5:1) three times to give the desired product (2.3 g, 87% purity). The solid was re-dissolved in DCM (50 mL), MTBE (50 mL) was added dropwise to above solution. The precipitate formed was collected by filtration and dried (1.8 g, 95% purity), which was then re-crystallized from THF (50 mL) to give the desired product (1.35 g, 98.8% purity) as a faint yellow solid: ESI $[M+H]^+$ = 436; ^{1}H NMR (400 MHz, $CDCl_3$) δ 8.53 (s, 1H), 7.62 – 7.53 (m, 4H), 7.27 (d, 1H, *J* =7.6 Hz), 7.24 (d, *J* = 1.9 Hz, 1H), 7.21 (d, *J* =1.9 Hz, 1H) 6.58-6.54 (m, 2H), 4.99 (s, 2H), 3.59 – 3.44 (m, 2H), 3.36 (td, *J* = 9.6, 6.9 Hz, 1H), 3.21 (t, *J* = 8.4 Hz, 1H), 2.98 – 2.83 (m, 1H), 2.34 (s, 6H), 2.30 – 2.22 (m, 1H), 2.08 – 1.91 (m, 1H).

Synthesis of TBX-2

4-(7-bromo-11-((2,2-dimethoxyethyl) amino)-5H-benzo[e]pyrrolo[1,2-a][1,4]diazepin-2-yl)benzonitrile (7)

To a solution of compound **6** (75 g 190 mmol, 1.0 eq), 2, 2-dimethoxyeth-anamine (100 g, 949 mmol, 5.0 eq) in THF/dioxane (500 mL/1L) was added. The resulting solution was stirred at 100 °C for 18 h. The mixture was concentrated under reduced pressure. The residue was re-dissolved in EA (300 mL), and HCl (1M, 1.2 L) was slowly added to above solution. A precipitate formed during the addition, which was collected by filtration. The filter cake was washed with EA several times and dried under vacuum to give the desired product (wet solid), which was used to the next step without further purification. ESI $[M+H]^+$ = 465.2/467.2

4-(7-bromo-9H-benzo[e]imidazo[2,1-c]pyrrolo[1,2-a][1,4]diazepin-12-yl)benzonitrile (Int-1)

To the solution of above solid, HCl (1M, 400 mL) in dioxane (800 mL) was added, and the resulting mixture was stirred at 80 °C for 20 h. The reaction mixture was cooled down to room temperature and filtered. The filter cake was washed with sat. $NaHCO_3$ solution (1 L) thoroughly followed by deionized water (1 L) wash. The solid was then slurried again with MeOH (300 mL). After filtration, the filter cake was collected and dried under vacuum to give the desired product as a white solid (36 g): ^1H NMR (400 MHz, DMSO-d_6) δ 7.92 (d, *J* = 2.3 Hz, 1H), 7.84 (d, *J* = 1.4 Hz, 1H), 7.75-7.774(m, 4H), 7.65 (d, *J* = 1.9 Hz, 1H), 7.56 (d, *J* = 8.6 Hz, 1H), 7.25 (d, *J* = 1.4 Hz, 1H), 7.06 (d, *J* = 1.9 Hz, 1H), 5.24 (s, 2H). ESI $[M+H]^+$ = 401.2/403.2

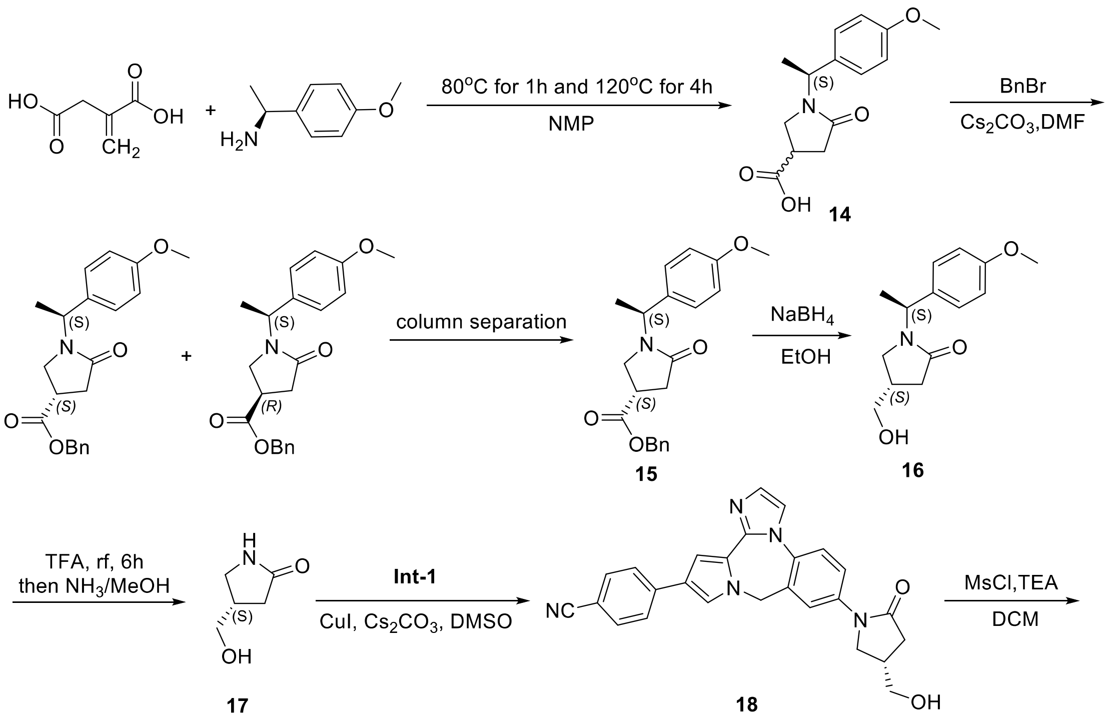

1-((S)-1-(4-methoxyphenyl)ethyl)-5-oxopyrrolidine-3-carboxylic acid (14)

A suspension of (S)-1-(4-methoxyphenyl) ethanamine (13.5 g, 99.15 mmol, 1.0 eq) and itaconic acid (13 g, 98.2 mmol, 0.9 eq) in 1-methyl-2-pyrrolidinone (80 mL) were heated to 80 °C for 1 h.

The solution was stirred for additional 4 hours at 120 °C. The reaction mixture was cooled to 25 °C and poured into 500 mL of demineralized water. The precipitate formed was filtered, washed with demineralized water and dried at 50 °C to give white solid (21 g, 79.8 mmol, 89% yield).

(S)-benzyl-1-((S)-1-(4-methoxyphenyl)ethyl)-5-oxopyrrolidine-3-carboxylate (15)

A solution of compound **14** (21 g, 79.8 mmol, 1.0 eq), benzyl bromide (10.34 mL, 87.8 mmol, 1.1 eq) and cesium carbonate (28.55 g, 87.8 mmol, 1.1 eq) in DMF (100 mL) were stirred at room temperature for 45 min. Water and ethyl acetate was added to the reaction mixture. The organic layer was separated and the aqueous layer was extracted with EtOAc (200 mL*2). The combined organic layers were dried over anhydrous Na_2SO_4 and concentrated under reduced pressure. The obtained residue was purified by silica gel column chromatography (solvent gradient: 5 percent to 50 percent petroleum ether / ethyl acetate) to give (S)- benzyl 1-((S)-1-(4-methoxyphenyl)ethyl)-5-oxopyrrolidine-3-carboxylate (11.2 g, 31.73mmol, 39.7% yield) as first eluent and (R)- benzyl 1-((S)-1-(4-methoxyphenyl)ethyl)-5-oxopyrrolidine-3-carboxylate (11.6 g, 32.86 mmol, 41.2% yield) as second eluent.

(S)- benzyl 1-((S)-1-(4-methoxyphenyl)ethyl)-5-oxopyrrolidine-3-carboxylate: ^{1}H NMR (400 MHz, $CDCl_3$) δ 7.42 – 7.28 (m, 5H), 7.20 (t, *J* = 5.7 Hz, 2H), 6.88 – 6.82 (m, 2H), 5.44 (q, *J* = 7.1 Hz, 1H), 5.14 (s, 2H), 3.79 (s, 3H), 3.53 (dd, *J* = 9.4, 5.8 Hz, 1H), 3.22 – 3.05 (m, 2H), 2.73 (qd, *J* = 17.1, 8.4 Hz, 2H), 1.48 (d, *J* = 7.1 Hz, 3H).

(R)- benzyl 1-((S)-1-(4-methoxyphenyl)ethyl)-5-oxopyrrolidine-3-carboxylate: ^{1}H NMR (400 MHz, $CDCl_3$) δ 7.38 – 7.30 (m, 3H), 7.27 – 7.22 (m, 2H), 7.18 (t, *J* = 5.7 Hz, 2H), 6.88 – 6.80 (m, 2H), 5.44 (q, *J* = 7.1 Hz, 1H), 5.06 (d, *J* = 1.5 Hz, 2H), 3.77 (d, *J* = 9.0 Hz, 3H), 3.58 – 3.47 (m, 1H), 3.28 – 3.14 (m, 2H), 2.80 – 2.61 (m, 2H), 1.49 (d, *J* = 7.1 Hz, 3H). ESI=354[M+H] and 354 [M+H].

(S)-4-(hydroxymethyl)-1-((S)-1-(4-methoxyphenyl)ethyl)pyrrolidin-2-one (16)

To a solution of compound **15** (11.2 g, 31.7 mmol, 1.1 eq), $NaBH_4$ (4.2 g, 111 mmol, 3.5 eq) in EtOH (75 mL) was added at 0 °C. The mixture was stirred at room temperature for 8 h. The

reaction was quenched by addition of NH_4Cl. EtOAc was added, and the organic layer was separated. The aqueous layer was extracted with EtOAc (3*200 mL). The combined organic layer was washed with brine, dried over anhydrous Na_2SO_4 and concentrated under reduced pressure. The resulting residue was purified by silica gel chromatography (PE: EA= 5:1 to ECM: MeOH=100: 1) to give the desired alcohol (7.5 g, 30.12 mmol, 95% yield). ESI: $[M+H]^+$ 250.

(S)-4-(hydroxymethyl) pyrrolidin-2-one (17)

A solution of compound **16** (7.5 g, 30.12 mmol, 1.0 eq) in TFA (40 mL) was heated to 80 °C and stirred for 16 h. Volatile solvents were removed under reduced pressure. The residue was re-dissolved in MeOH (50 mL), and $NH_3{\cdot}H_2O$ was added to above solution at room temperature. After stirring for 1h, the reaction mixture was concentrated, and the residue was purified by silica gel column chromatography (DCM: MeOH = 100:1 to 10:1) to give the desired product (3.0 g, 26.1 mmol). ^1H NMR (400 MHz, MeOH-d_4) δ 3.58 – 3.51 (m, 2H), 3.51 – 3.45 (m, 1H), 3.20 (dd, J = 10.1, 5.3 Hz, 1H), 2.72 – 2.56 (m, 1H), 2.42 (dd, J = 17.1, 9.1 Hz, 1H), 2.24 – 2.05 (m, 1H). ESI= $[M+H]^+$ 116

(S)-4-(7-(4-(hydroxymethyl)-2-oxopyrrolidin-1-yl)-9H-benzo[e]imidazo[2,1-c]pyrrolo[1,2-a][1,4]diazepin-12-yl)benzonitrile (18)

To the solution of **Int-1** (26.9 g, 67.19 mmol, 1.0 eq), compound **17** (8.5 g, 73.91 mmol, 1.1 eq) in dry DMSO (270 mL), CuI (6.4 g, 33.60 mmol, 0.5 eq), *trans*-N^1,N^2-dimethylcyclohexane-1,2-diamine (9.6 g, 67.19 mmol, 1.0 eq) and K_3PO_4 (28.5 g, 134.38 mmol, 2.0 eq) were added. The reaction system was purged with argon gas and stirred at 115 °C for 18 h. The reaction mixture was cooled down to room temperature and diluted with DCM/MeOH (10:1, 300 mL). The resulting mixture was filtered, and the filtrate was concentrated. Water (1.5 L) was added slowly. The solid formed was collected by filtration, washed with excess water and dried under high vacuum. The

crude product was then passed through a short pad of silica gel column with DCM and MeOH as eluent to give the desired product (28.7 g, yield: 98.2%) as an off-white solid. ESI $[M+H]^+$ = 436.

(S)-(1-(12-(4-cyanophenyl)-9H-benzo[e]imidazo[2,1-c]pyrrolo[1,2-a][1,4]diazepin-7-yl)-5-oxopyrrolidin-3-yl)methyl methanesulfonate

To a suspension of the above intermediate (28.7 g, 66.0 mmol, 1.0 eq), MsCl (6.1 mL, 79.2 mmol, 1.2 eq) and TEA (13.6 mL, 99.0 mmol, 1.5 eq) in dry DCM (290 mL) were added dropwise at 0 °C. The resulting mixture was stirred at 0 °C for 0.5 h. The reaction was quenched with sat. aqueous $NaHCO_3$ and then extracted with DCM/MeOH (10:1, 300 mL X 3). The combined organic layers were washed with brine (100 mL), dried over Na_2SO_4, filtered and concentrated. The resulting residue was purified by silica gel column (DCM: MeOH = 20:1) to give the desired product (31.6 g, yield: 93.4%) as an off-white solid. ESI $[M+H]^+$ = 514.

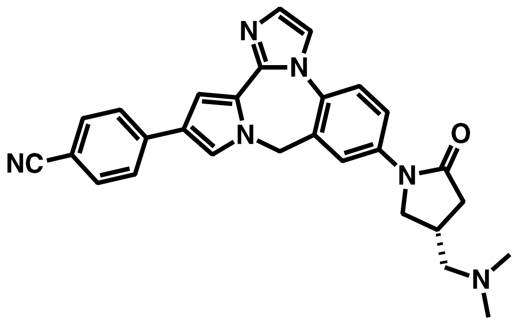

(R)-4-(7-(4-((Dimethylamino)methyl)-2-oxopyrrolidin-1-yl)-9H-benzo[e]imidazo[2,1-c]pyrrolo[1,2-a][1,4]diazepin-12-yl) benzonitrile (TBX-2)

To a suspension of the above intermediate (26.5 g, 51.66 mmol), dimethylamine (2.0 M in THF, 129 mL, 258.28 mmol) in dry DMF (100 mL) was added. The resulting mixture was stirred at 100 °C for 18 h in sealed tube. The reaction mixture was concentrated under reduced pressure. To the residue above, water (500 mL) was added slowly. A solid precipitate formed, which was collected by filtration and washed with excess water, and then dried. The crude product was

triturated with THF (200 mL), filtered, and dried under high vacuum to give the desired product (13.8 g, purity: 98%, yield: 58%) as an off-white solid: ESI $[M+H]^+$ = 463; ^{1}H NMR (400 MHz, MeOH-d_4) δ 7.97 (d, *J* = 2.5 Hz, 1H), 7.78 (dd, *J* = 8.8, 2.5 Hz, 1H), 7.74 – 7.67 (m, 3H), 7.66 – 7.62 (m, 2H), 7.58 (d, *J* = 8.8 Hz, 1H), 7.53 (d, *J* = 1.9 Hz, 1H), 7.25 (d, *J* = 1.3 Hz, 1H), 7.05 (d, *J* = 1.9 Hz, 1H), 5.18 (s, 2H), 4.05 (dd, *J* = 9.7, 7.8 Hz, 1H), 3.76 – 3.67 (m, 1H), 2.83 – 2.71 (m, 2H), 2.51 (dd, *J* = 12.3, 7.7 Hz, 1H), 2.47 – 2.37 (m, 2H), 2.30 (s, 6H).
